# Analyzing the Trends of COVID-19 and Human Activity Intensity in Malaysia

**DOI:** 10.3390/tropicalmed8020072

**Published:** 2023-01-19

**Authors:** Wei Chien Benny Chin, Chun-Hsiang Chan

**Affiliations:** 1 Department of Geography, National University of Singapore, Singapore 117570, Singapore; 2 Undergraduate Program in Intelligent Computing and Big Data, Chung Yuan Christian University, Taoyuan City 320314, Taiwan

**Keywords:** Malaysia, COVID-19, human activities, travel restriction, travel willingness, time series

## Abstract

COVID-19 has struck the world with multiple waves. Each wave was caused by a variant and presented different peaks and baselines. This made the identification of waves with the time series of the cases a difficult task. Human activity intensities may affect the occurrence of an outbreak. We demonstrated a metric of time series, namely log-moving-average-ratio (LMAR), to identify the waves and directions of the changes in the disease cases and check-ins (MySejahtera). Based on the detected waves and changes, we explore the relationship between the two. Using the stimulus-organism-response model with our results, we presented a four-stage model: (1) government-imposed movement restrictions, (2) revenge travel, (3) self-imposed movement reduction, and (4) the new normal. The inverse patterns between check-ins and pandemic waves suggested that the self-imposed movement reduction would naturally happen and would be sufficient for a smaller epidemic wave. People may spontaneously be aware of the severity of epidemic situations and take appropriate disease prevention measures to reduce the risks of exposure and infection. In summary, LMAR is more sensitive to the waves and could be adopted to characterize the association between travel willingness and confirmed disease cases.

## 1. Introduction

COVID-19 presented to the world how a highly contagious disease could strike, i.e., by forming multiple waves of outbreaks that were caused by multiple variants of a virus, some happening within overlapping periods. These waves include the first COVID-19 wave that started in January 2020 and led to the worldwide lockdown situation from March 2020 [1,2], the Delta wave that started from late 2020 and spread globally by late 2021 [3,4], followed by Omicron and its subvariants which happened around November 2021 to the end of 2022 [5,6]. Although it was a question of the gene and strain of the virus, the waves of COVID-19 also raised a spacetime question of when each wave started and stopped in each country or city [7]. Given the situation that variants can spread to a city at any time and surge the cases at different speeds, several waves of the disease overlap with each other. The overlapping waves led to two situations: the waves of cases were never dropped to the baseline (zero cases) ever since the first wave of COVID-19, which indicated that the baselines were changing for different variants; the waves could have different peak values, e.g., the peak of the first wave may be low compared to the Omicron variant(s). Therefore, it is difficult to differentiate the waves and identify the time of beginning and end of each wave for a city. It is even more difficult to compare between places due to the differences in population size and magnitude of cases.

Time series models had been used in the analysis of COVID-19 studies, including the autoregressive integrated moving average model [8,9], statistical neural network model [10], long short-term memory model [11] and Gaussian process regression model [12]. A concept from the analysis of the trading indicator—moving average convergence/divergence (MACD)—can be adopted. MACD is an indicator comparing the short-term moving average (MA) to the long-term trend (MA) [13]. The crossings—also known as golden and death crosses—indicate the upward and downward trends (respectively) of the short-term moving average crossing the long-term moving average values. In other words, the long-term moving average value shows the relatively stable and balanced level of the trend line, whereas the short-term moving average represents the current tendency of the situation. Thus, the upward and downward crosses of the short-term trend line on the long-term trend line raise the signals of important changes in the situation. Since the COVID-19 waves have a different baseline, the comparison between short-term MA and long-term MA can show a pattern that presents the up and down trending of the recent situation. This is because short-term MA is more sensitive to the recent development of the situation, whereas long-term MA can reflect the new baseline of the cases. Thus, comparing short-term and long-term MAs can hint at when the disease cases begin to surge and drop.

The conventional MACD calculates the absolute difference between the exponential MA of short-term and long-term trends. However, the waves present different highest points. The magnitude of the peak would make the absolute difference between the short-term/long-term MAs vary between waves. This indicated that the magnitudes of the MACD would be different between the waves of the various COVID-19 variants. Whereas this may be useful for comparing the waves in absolute numbers, e.g., the first wave may be less contagious than the later waves, it may also raise difficulties in the observation of the disease situation in terms of the local maximum, e.g., the first wave may be flattened when compared with the Omicron wave and give an impression that the earlier waves were less severe than the latter. Thus, an alternative way to compare the short-term/long-term MAs is needed to capture the trends and present the changes in pandemic situations. However, the state-of-the-art methods could not identify the increase and decrease period because these methods concentrated on time series disease prediction. In this study, we presented a variant of the MACD to analyze the disease trends and identify the waves of the disease using an alternative comparison approach, namely the log moving average ratio (LMAR). A detailed description is presented in the Section 2.

To analyze and demonstrate the proposed analysis framework, in this study, we presented a series of analyses using the Malaysian COVID-19 situation as a case study. COVID-19 started in Malaysia with the first wave in January 2020. The first case occurred when three Chinese citizens traveled to Malaysia from an international conference held in Singapore where they were infected [14,15]. Although these early cases did not lead to a superspreading situation, many imported cases continued to appear in Malaysia over the next two months, which eventually led to a rapid increase of the community cases caused by a mass local gathering and superspreading event [16]. The high number of cases forced the government to impose a nationwide lockdown in March 2020—the Malaysian Movement Control Order (MCO) [17]. The MCO marked the starting point of a series of ups and downs of the disease cases and the changes in the human activity of the pandemic in Malaysia, including several phases of the National Recovery Plan (NRP) and a series of state-level or national-level lockdown measures until April 2022 [15,18,19].

During the pandemic, the Malaysian government introduced a human activity intensity monitoring scheme named MySejahtera [20]—a mobile application that requires people to scan a QR code before entering any premises (shops, offices, buildings, etc.). The check-in (QR code scanning) process was compulsory during the pandemic starting from around August 2020 to April 2022 [21,22]. In other words, this monitoring scheme recorded the number of people entering every premise and the time series of the check-ins indicates the travel willingness of different phases of NRP or MCO. Indeed, the multiple waves of COVID-19 and its variants/subvariants had made every city in the world experience several instances of movement restrictions imposed by the government [15,18,19] or self-imposed movement reduction [23,24]. Starting from the first lockdown of COVID-19, people tend to stay at home and reduce their outdoor activities, including work, social gatherings, shopping, or exercising, to reduce physical interactions and close contact. Although this is an effective way of self-protecting in the early phases, it also reduces the travel willingness of people and changes their habits as a short-term or even a long-term effect [25,26]. Understanding the relationship between traveling willingness and pandemic waves could facilitate the policy-making process for epidemic control. However, present methods considering both the short-term and long-term impacts of pandemic waves on travel willingness are limited.

This study aims to identify the waves of disease and human activity intensity in Malaysia. This study focuses on two objectives: (1) to demonstrate how the waves of the disease cases and the check-ins can be identified, and (2) if there is a relationship between the disease situation and human activity intensity. Previous studies showed that human activity intensity and disease cases were positively correlated [27,28,29]. This means that when a place has a higher intensity of human activity, it may lead to a more serious outbreak. However, from the time series analysis perspective, a time lag must exist between the human activity intensity and the outbreak. During an outbreak of the disease, government-imposed movement restrictions or self-imposed travel willingness would reduce human activity intensity. This indicated that the outbreak would be followed by a reduction in human activity intensity as well [30,31]. Therefore, we would explore the patterns of the disease cases and the check-ins to uncover the relationship between the two sets of time series.

## 2. Materials and Methods

**Figure 1:**
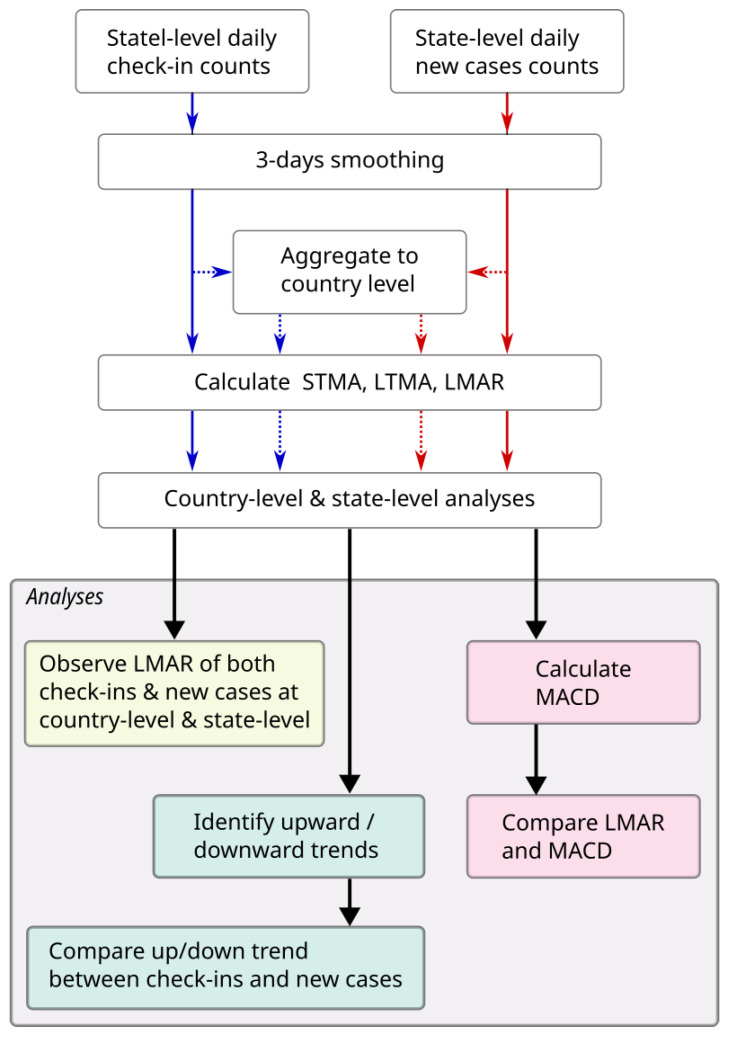
The flowchart of this study.

The flowchart of the calculation and analysis were shown in Figure 1.

### 2.1. Study Area and Study Period

**Figure 2:**
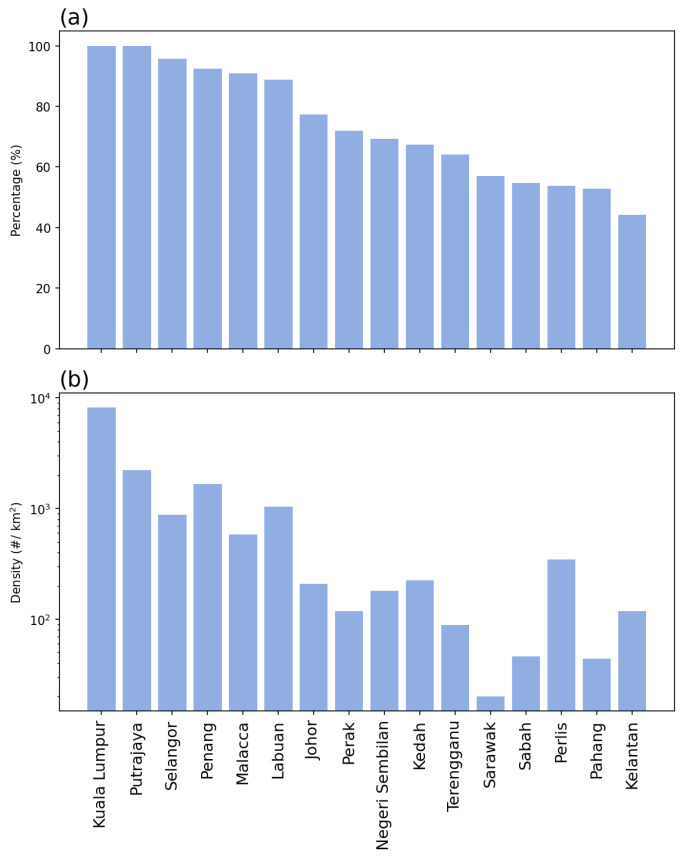
Demographics of Malaysia: (**a**) urban population percentages, and (**b**) population density.

The study area was set to be Malaysia, a Southeast Asian country composed of two parts: West Malaysia (also known as Peninsular Malaysia, which shares a border with Thailand in the north and is connected to Singapore across the Johor Strait), and East Malaysia (part of the island of Borneo). In this study, two spatial levels were analyzed, including the country level and the states and federal territories (hereafter, state level). A total of 16 states or federal territories (FTs) are in Malaysia, 13 of which are located in West Malaysia: on the west coast, from north to south: Perlis (PL), Kedah (KH), Penang (PG), Perak (PK), Selangor (SL), FT Kuala Lumpur (KL), FT Putrajaya (PJ), Negeri Sembilan (NS), Malacca (ME), and Johor (JH); on the east coast, Kelantan (KN), Terengganu (TE), and Pahang (PH). The other three states are located in East Malaysia, including Sabah (SA), Sarawak (SK), and FT Labuan (LA). Figure 2 shows the demographic information, including the urban population percentage and population density of the 16 states, sorted by the urban population percentages [32].

**Figure 3:**
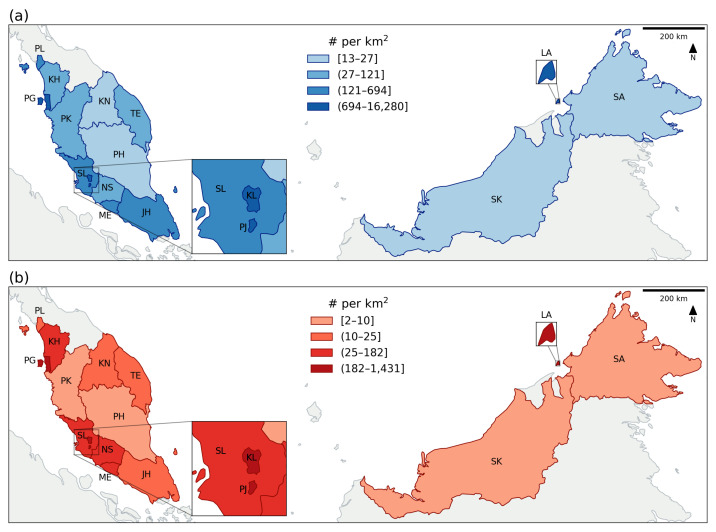
The spatial distribution of (**a**) the daily average check-in counts, and (**b**) the total COVID-19 cases within the study period. The area code can refer to the study area and study period section.

In terms of temporal range, due to the availability of data for human activities, the data used in this study were from 1 December 2020 to 30 April 2022 (a total of 17 months). The human activity data were acquired from the government-published open data repository, provided by the Ministry of Health, Malaysia (repository url: https://github.com/MoH-Malaysia/covid19-public, accessed on 28 October 2022) [33]. Starting from April 2020, the Malaysian government launched the MySejahterah app, which was a requirement for people when they leave their homes [20]. People were required to scan a QR code (check-in) when entering any premises or buildings during the pandemic for the purpose of crowd control and data recording for the continuous development of disease control measures starting from August 2020 [21]. There was another function of the app, which recorded the proximity of people through the nearby Bluetooth signal recording, but the data were not released due to privacy concerns. Because the analysis involved moving average settings (as described below), the first two months were ignored in the visualization, and the remaining analysis period was between 1 February 2021 and 30 April 2022 (15 months). Figure 3 shows the daily average check-in density for each state and the total density of COVID-19 within the 17 months of the study period.

### 2.2. Check-Ins and Disease Datasets

**Figure 4:**
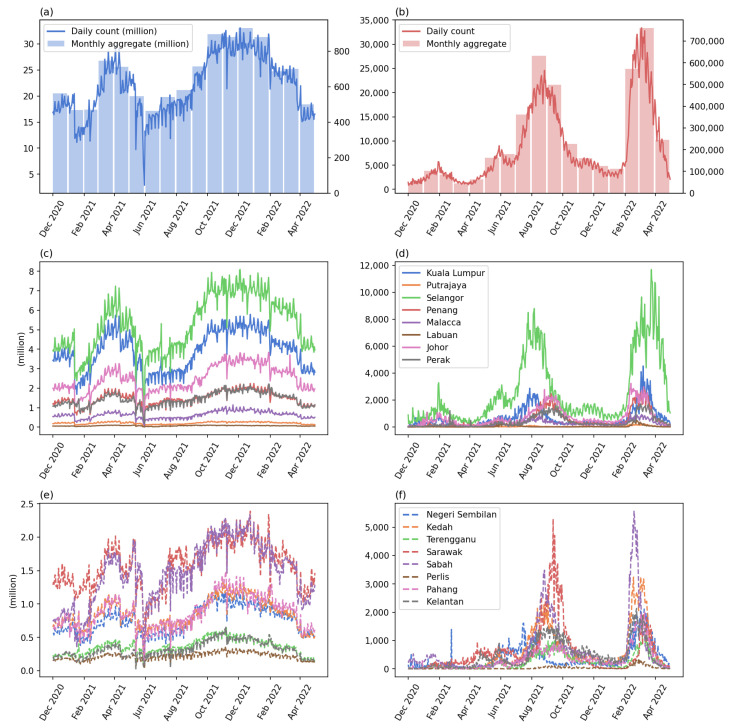
The time series of the check-ins and new case counts: (**a**) the daily count (blue line, left axis) and monthly aggregated (blue bars, right axis) of check-in count, (**b**) the daily count (red line, left axis) and monthly aggregated (red bars, right axis) of COVID-19 case count, (**c**,**e**) the check-in counts of the first and second eight states sorted by the urbanized population percentage, (**d**,**f**) the COVID-19 cases of the two groups of states. The X-axis shows the months starting from 1 December 2020 to 30 April 2022.

As aforementioned, the datasets were provided by the Ministry of Health, Malaysia under an open data license (https://github.com/MoH-Malaysia/covid19-public, accessed on 28 October 2022). Two main datasets were used: (1) the daily check-in counts for each state and (2) the daily new case count for each state. Both datasets were publicly available on the aforementioned repository. Figure 4 shows the daily or monthly trend of the aggregated and individual state count of the two datasets. From the right column (new case count), the two waves of COVID-19 can be observed, the Delta variant that peaked in August 2021 and the Omicron variant that peaked in March 2022.

### 2.3. Data Preprocessing

Because the confirmation of COVID-19 cases includes some uncertain time delay, e.g., the influence of weekends and reporting delays, in this study, we used a simple moving average of 3 days to smooth each of the original COVID-19 new case time series (Equation (1)). For consistency purposes, the check-in time series was also smoothed using the same equation. The smoothing process also helped fill in missing data in the check-in counts (27–31 May 2021). In the equation, ${\mathrm{val}}_{t}$ is the value of day-*t*, *x* is the number of days before *t*, and ${\mathrm{raw}}_{t-x}$ is the original value of the time series at *x* days before day-*t*. When x=0, it is the current day. This moving average will sum the three days before and including the current day, and divide the summation by 3.

(1) $${\mathrm{val}}_{t}=\frac{1}{3}\sum_{x=0}^{2}{\mathrm{raw}}_{t-x}$$

### 2.4. Analysis and Calculations

The main purpose of the study is to identify the temporal trend of the two dimensions, that is, the dynamic of human activity intensity and the disease outbreak situation. To measure the temporal trend, this study proposed a metric called log moving average ratio (LMAR) for the identification of different waves from the daily time series. The main difficulty of identification is caused by the overlap of multiple outbreak waves: the next wave strikes again before the previous wave ends, and the shapes and peaks of the waves were different within the same study area or between different places.

In a disease outbreak context, when the first wave strikes for some time, both short-term and long-term patterns increase; whereas the long-term trend slowly increases, the short-term trend is more sensitive and reflective of the recent development. Therefore, as the outbreak started to reduce, the short-term trend will start dropping, and when it goes below the long-term trend, this provides a signal of recovery from the first wave. If the second wave starts at the study area during the recovery of the first wave, the starting point of the second wave may be above the zero line because the first wave does not end. Therefore, it is hard to differentiate the two waves of outbreaks. In this situation, a MACD-like metric can be useful. MACD compares the short-term trend with the long-term trend and focused on identifying the crosses—upward cross when the short-term trend crosses from below to above the long-term trend, and downward cross when it goes down and below the long-term line. In other words, because the first wave of outbreaks raises the long-term trend line, the increasing short-term trend will only need to compare the current situation with the long-term pattern—if the short-term trend goes beyond the long-term trend, it signals the second wave of outbreak.

In this study, we proposed that LMAR is more suitable for the waves of disease outbreaks. LMAR is similar to MACD in terms of comparing a short-term pattern with a long-term trend, but LMAR focuses more on the magnitude of changes rather than the absolute differences in sizes. The calculation involved four steps: (1) short-term moving average (STMA), (2) long-term moving average (LTMA), (3) LMAR, and (4) standardization.

STMA captures the recent situations. The 7-day pattern can present a short-term change which is sufficient to capture the immediate changes of the time while not being influenced by the random fluctuation that may be caused by the delayed reporting or weekend lags [34,35]. Therefore, the STMA calculation used 7-day values (inclusive of the target day) to calculate the simple moving average (Equation (2)).

(2) $${\mathrm{STMA}}_{t}=\frac{1}{7}\sum_{x=0}^{6}{\mathrm{val}}_{t-x}$$

LTMA captures the longer pattern up to the present day (Equation (3)). In this study, we used 56 days (8 weeks) to measure the long-term pattern of the data. A previous study observed that a typical wave of the disease could last for 4 months [36]. During the time period of a wave, usually the first half presented an increasing trend and was followed by a decreasing trend in the second half. The incubation period of COVID-19 was around 8.2 to 15.6 days (approximately 1 to 2 weeks) [37] with an infectious period of around 2–3 days before and after the onset of symptoms [38]. In other words, 2 months indicates half of a wave, which is about 4 to 8 rounds of infections. As such, we used a 56-day pattern to capture the long-term condition of the outbreaks, which serves as a baseline in the LMAR calculation.

(3) $${\mathrm{LTMA}}_{t}=\frac{1}{56}\sum_{x=0}^{55}{\mathrm{val}}_{t-x}$$

LMAR is calculated as the log of the ratio of STMA to LTMA (Equation (4)). The ratio of the two moving averages indicates the times of differences between the two values. For example, if the short-term is two times greater than the long-term moving average, the ratio would be equal to two, whereas if the short-term is two times less than the long-term, it would be 1/2. Using these two situations as an example, taking a log with base 2 will convert the ratio into +1 and −1, respectively. When the short-term is equal to the long-term pattern, the ratio equals 1 and the LMAR equals 0.

(4) $${\mathrm{LMAR}}_{t}=\log_{2}\frac{{\mathrm{STMA}}_{t}}{{\mathrm{LTMA}}_{t}}$$

### 2.5. Study Design

In this study, two scales of analysis were performed, i.e., the country level (Malaysia as a case study) and the state level (16 states, each as a case study). For each of the case studies, LMAR was calculated for both daily check-in counts and new case counts, and the two calculation results were compared to discuss the relationship between human activity intensity and the disease situation. Following the analyses of the two different scales, we performed the two comparison analyses, including the comparison with MACD and the comparison of the up and down trends between the case counts and check-in counts. In this study, for consistency purposes, the MACD was calculated as Equation (5), which used the 7-day and 56-day simple moving averages instead of the 12-day and 26-day exponential moving averages.

(5) $${\mathrm{MACD}}_{t}={\mathrm{STMA}}_{t}-{\mathrm{LTMA}}_{t}$$

The up and down trends of a time series can be identified based on the LMAR values, i.e., a value of greater than zero indicates that the wave presents an upward trend, whereas a less than zero value indicates a downward trend (see Equation (6)). The trend can be considered static if the LMAR equals zero, which occurs only when the STMA is equal to LTMA. The underlying implication of this is that a positive value of LMAR implies that the STMA is higher than LTMA, which presents an increasing trend, and vice versa for a negative value. For each state/FT, we calculated the percentage of days for the four situations: (1) both check-ins and new cases were increased (up–up), (2) the new case count increased but check-ins decreased (up–down), (3) new cause count decreased but check-ins increased (down–up), and (4) both check-ins and new case count decreased (down–down). The percentages of days when both new case count and check-in count are in the same direction (up–up or down–down) and in the opposite directions (up–down or down–up) are also calculated.

(6) $${\mathrm{trend}}_{t}=\left\{\begin{array}{cc} \mathrm{upward} & ,\;\mathrm{if}\;{\mathrm{LMAR}}_{t}>0,\\ \mathrm{static} & ,\;\mathrm{if}\;{\mathrm{LMAR}}_{t}=0,\\ \mathrm{downward} & ,\;\mathrm{if}\;{\mathrm{LMAR}}_{t}<0, \end{array}\right.$$

## 3. Results

### 3.1. Country Level Analysis

**Figure 5:**
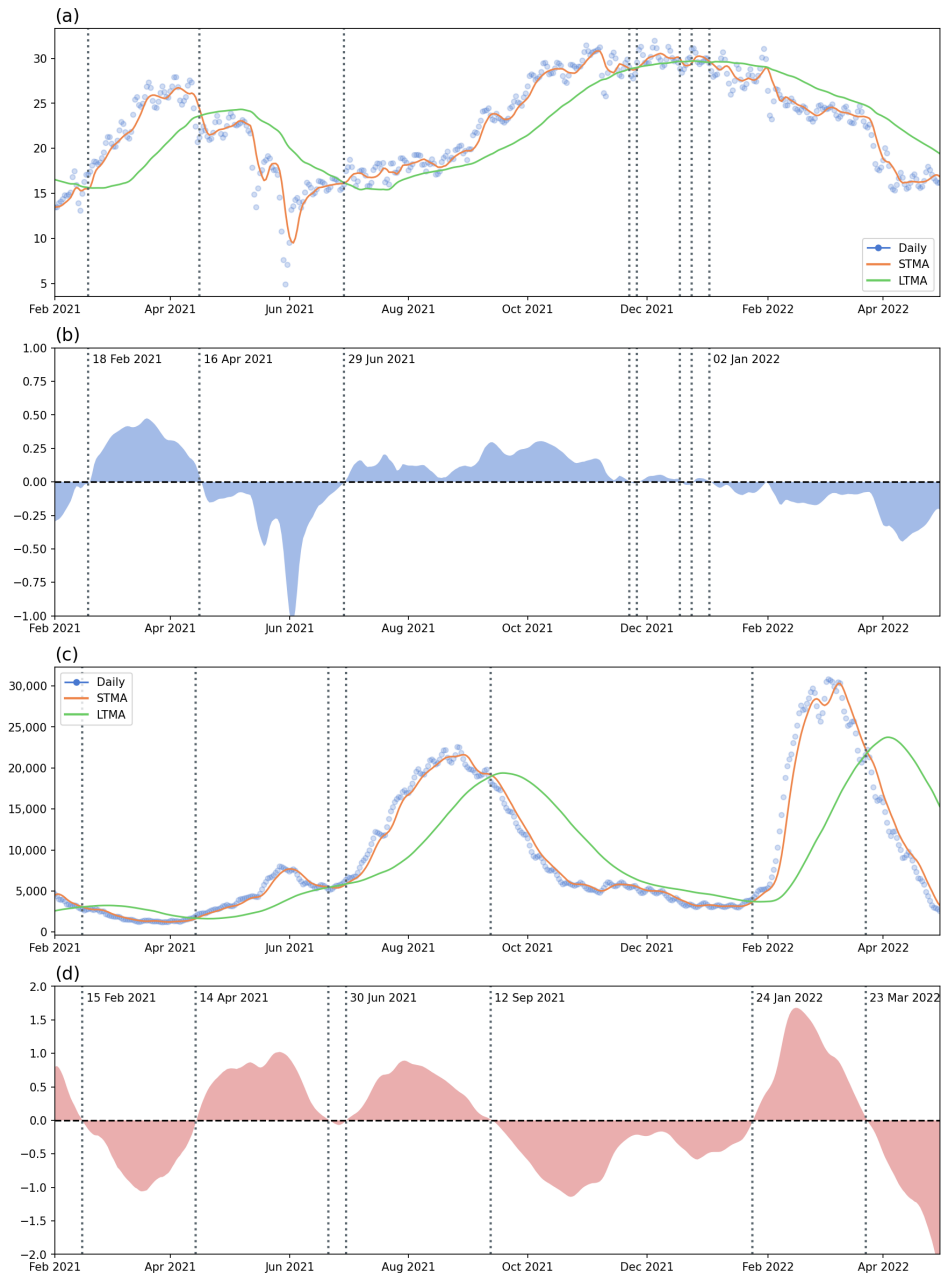
The time series (with short-term/long-term moving average) and the LMAR: (**a**) the check-in counts and moving averages, (**b**) the LMAR of check-ins, (**c**) the new case count and moving average, and (**d**) the LMAR of new case count. The dates of the significant short-term/long-term crossing for the two (check-ins and COVID-19 cases) were written in the LMAR figures (vertical lines).

In the first analysis, the two datasets were aggregated at the country level. Figure 5 shows the data and results of the check-in counts and new COVID-19 confirmed case count. Figure 5a presents the daily counts of check-in datasets (points), the STMA (red line), and the LTMA (green line). Figure 5b shows the LMAR of the check-ins with the dates highlighting the crossing with the horizontal line (LMAR = 0). The horizontal line of zero indicated the crossing of the STMA and LTMA lines. If the LMAR is greater than zero, it means that the STMA is greater than LTMA, and vice versa. Thus, the crossing from above to below highlights the beginning time point of the decreasing trend, and the crossing from below to above the horizontal line indicates the starting date of the increasing trend. These crossings can also be observed in Figure 5a. Similarly, Figure 5c presents the data, STMA, and LTMA of the daily new COVID-19 confirmed case count in Malaysia during the study period, whereas Figure 5d shows the LMAR of the new case counts.

Regarding the check-in counts patterns, there is a clear increasing pattern in the early stage of the study period, followed by a decreasing trend since 16 April 2021. The decrease in check-ins may be caused by the first increasing wave of COVID-19 cases that started on 14 April 2021. The daily number of cases reached a peak value of around 10,000 in June 2021; after a small decrease, the trend continue to increase from the end of June 2021—a wave caused by the Delta variant. However, at the same time, the check-ins data indicated a slow but stable increment trend. The Delta wave started to decrease on 12 September and reached a relatively low daily new case count (around 5000) at the end of 2021. There were a lot of small crossing events that occurred in December 2021 and January 2022, indicating an unstable situation during the two months. This may be caused by the check-ins and human activity intensity having reached peak values, and also the sense of security due to the relatively low number of COVID-19 cases.

Since the beginning of 2022, a new wave of COVID-19 cases started on 24 January 2022. This was the first Omicron wave striking Malaysia. As a response to the outbreak, human activity started to decrease until the end of the study period. Based on the daily new case counts, the first Omicron wave started to decrease on 23 March 2022 until the end of the study period.

Through the analysis using the country-level aggregated data, we can observe that the two datasets, namely the human activity intensity (check-ins) and the disease situation (newly confirmed case count), were mostly reversed or influencing one another. For example, the increment in new cases may lead to a decrement in check-ins and vice versa. On the other hand, this analysis also demonstrated that the two waves of the disease and the recovery or responses of people in terms of outdoor activities can be identified from the LMAR plots.

### 3.2. State-Level Analysis

**Figure 6:**
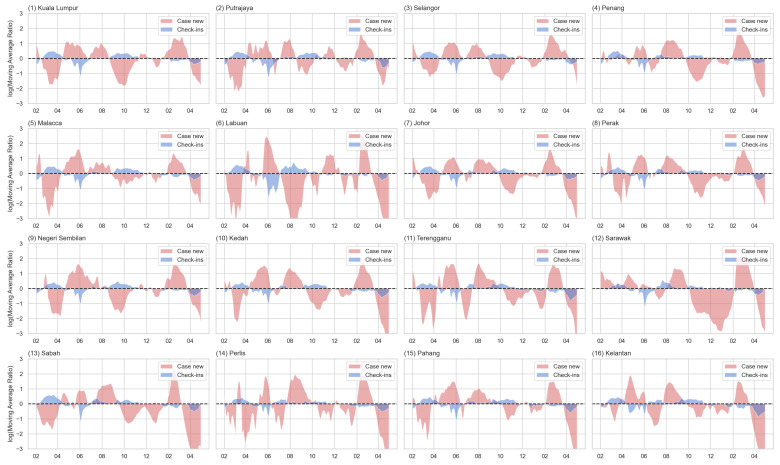
The LMAR of the check-in counts (blue) and new case counts (red) for each state (sorted by the urbanized population percentage).

**Figure A1:**
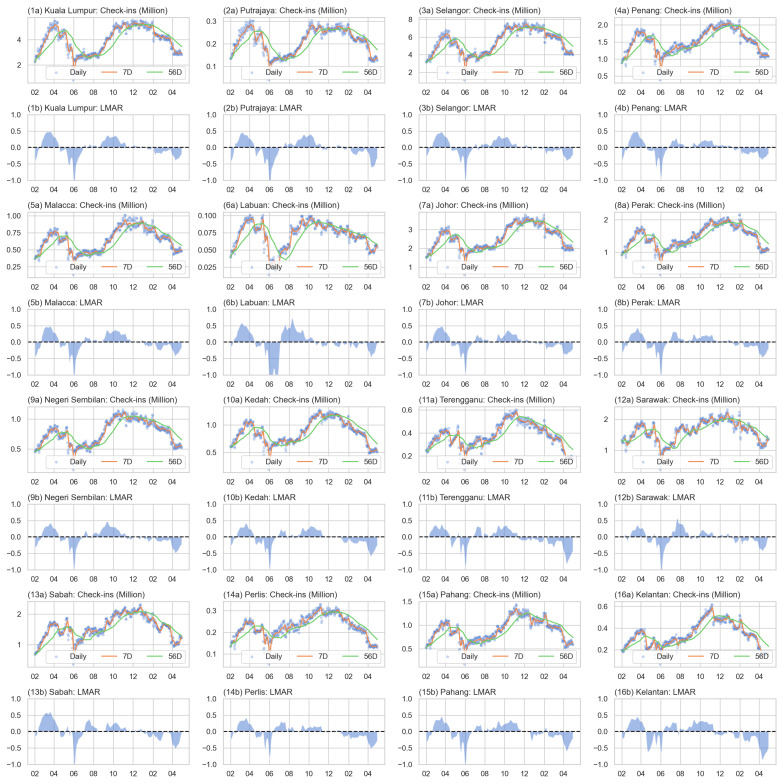
The state-level analysis for the check-in counts. The odd rows (1st, 3rd, 5th, 7th) show the time series (in millions) with short-term (7 days) and long-term (56 days) moving averages of each state (sorted by the urbanized population percentage). The even rows (2nd, 4th, 6th, 8th) show the LMAR based on the time series above.

**Figure A2:**
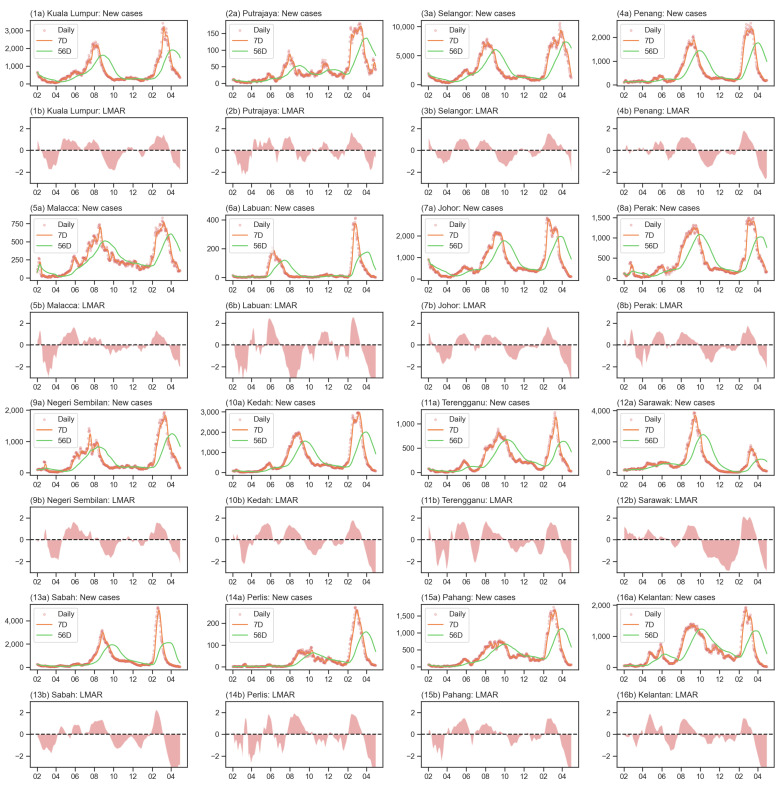
The state-level analysis for the new case counts. The odd rows (1st, 3rd, 5th, 7th) show the time series with short-term (7 days) and long-term (56 days) moving averages of each state (sorted by the urbanized population percentage). The even rows (2nd, 4th, 6th, 8th) show the LMAR based on the time series above.

In this section, the 16 states were used as an independent study case. Figure 6 shows the LMAR of the check-in counts (blue) and new case count (red) for each state. The detailed comparison between input data, STMA, LTMA, and LMAR was presented in Appendix A, Figure A1 and Figure A2. Similar to the country-level analysis, the state pattern also indicated a complementary relationship between the two datasets, especially in the year 2021. This indicated that the two trends were opposite of each other. This pattern means that the two datasets were negatively correlated. In other words, with slight time differences, when the case count increases, human activity intensity tends to decrease, and when the case count decreases, the human activity will also start to increase. This was especially the case in the year 2021. For example, in most states, there was an increase in check-ins from the beginning with a decreasing trend in the case counts. This wave of increasing human activity intensity was followed by an increasing trend of new case counts with a decrease in check-ins starting from April 2021. Some of the states contained a decreasing trend of disease cases with the increment of human activity from July to October 2021.

### 3.3. The Comparison of LMAR and MACD

**Figure 7:**
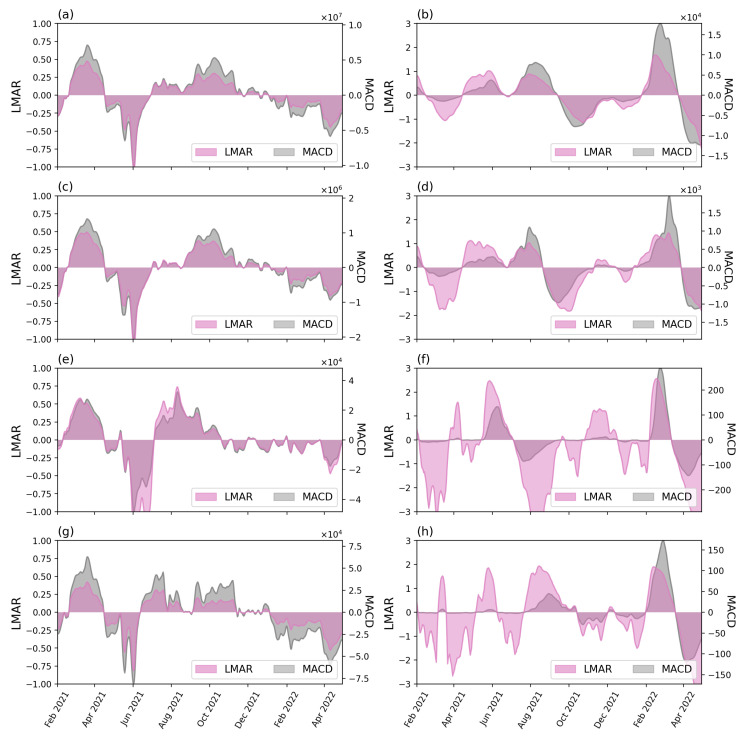
The LMAR (magenta) and MACD (grey) of the check-in counts (left) and new case counts (right column) for the country (top row, (**a**,**b**)) and three selected states: (2nd row, (**c**,**d**)) Kuala Lumpur, (3rd row, (**e**,**f**)) Labuan, and (4th row (**g**,**h**)) Perlis.

**Figure A3:**
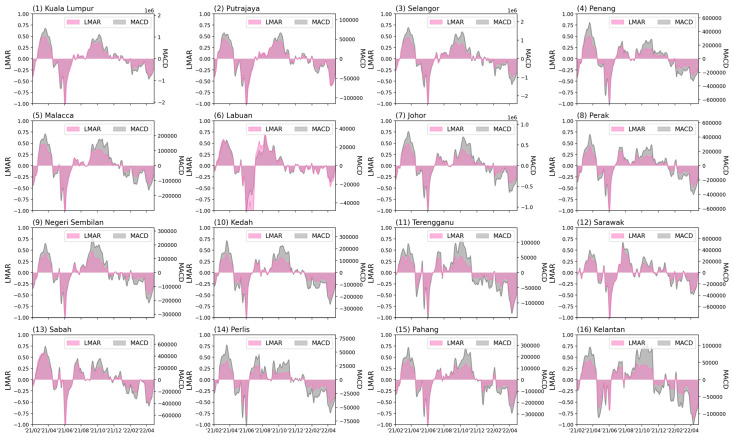
The comparison between LMAR and MACD of check-in count time series for each of the 16 states/FTs.

**Figure A4:**
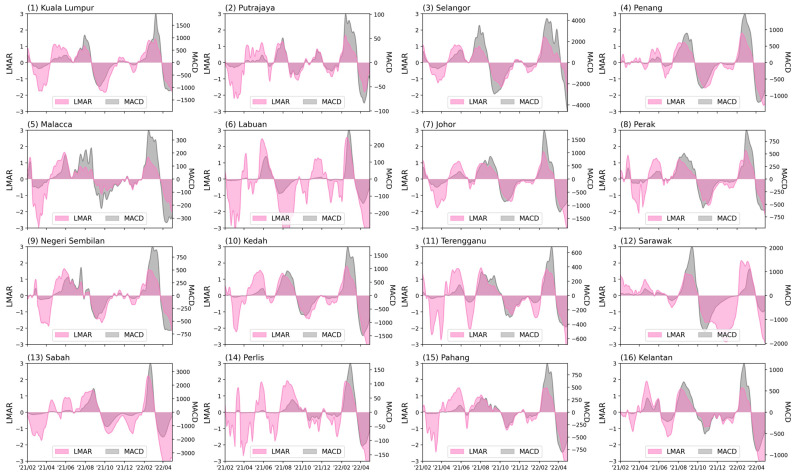
The comparison between LMAR and MACD of new case count time series for each of the 16 states/FTs.

To understand the differences between LMAR and the conventional MACD, we presented a comparison analysis between the two indexes. Figure 7 shows the LMAR (magenta color, values labeled on left axis) and MACD (grey color, values labeled on the right axis) of the two sets of data for the country level and three selected states/FTs. The complete comparisons of the 16 states/FTs were shown in Appendix A (Figure A3 and Figure A4).

Overall, LMAR and MACD yield similar trends in each case for both check-in counts and new case counts. The differences between LMAR and MACD are in the values. MACD calculated the actual differences between the STMA and LTMA trends (e.g., the short-term situation has 1000 cases more than the long-term situation), whereas LMAR focused on the magnitude of differences (e.g., the short-term situation has doubled the size of the long-term). The actual differences can vary from time to time and between case studies. This led to the situation that for some cases, some small value MACD waves were not visible because another period contained a large wave where the actual differences were several times larger than the previous small waves, e.g., the early waves of the new case counts at the four selected cases presented in Figure 7 (right column), while comparing across different study cases, the maximum and minimum MACD values (vertical axis) can be different from hundreds to tens of millions. These made it difficult and inconvenient when comparing various study cases. On the other hand, the relative differences (LMAR) show more stable values, i.e., between ±1 or between ±3 in this study. The relative differences provide a better visual effect for the highlighting of the waves. Take the new case count of the Perlis as an example; the case count of the earlier wave (Delta) is visible with LMAR but not visible with MACD. The values of the LMAR was a useful index to show the magnitude of the relative differences, e.g., −1 and 1 indicates that the short-term value is, respectively, a half ($2^{-1}$) or double ($2^{1}$) of the long-term value, ±2 indicates the relative differences is $2^{-2}$ or $2^{2}$×, etc.

### 3.4. The Comparison of Upward and Downward Trends of the Check-In Counts and Case Counts

**Figure 8:**
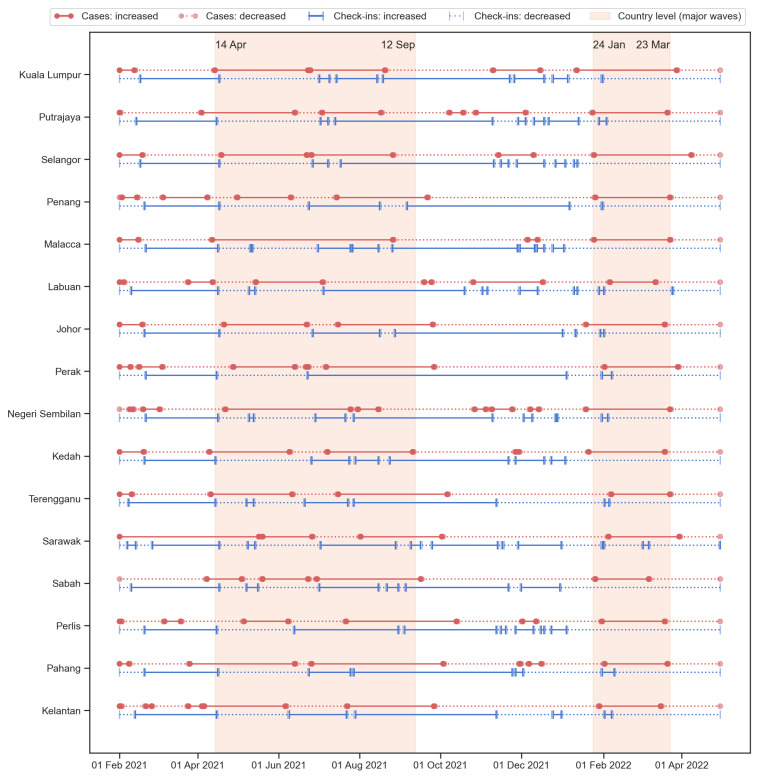
The temporal range of increasing (solid lines) and decreasing (dotted lines) trends for the new cases (red color with circle endpoints) and check-ins (blue color with ticker endpoints). The two shaded areas indicated the two waves according to the country-level analysis, i.e., the Delta wave occurred in 2021 and the first Omicron wave occurred in early 2022.

Figure 8 shows the increased and decreased time during the study period. Based on the temporal range plot, the starting/ending dates of various waves of disease were similar between states. This is especially the case for the Omicron wave. The country-level results for Delta and Omicron waves were also shown in the figure. The temporal range of the Delta variant was slightly longer. Based on Figure 5c and Figure A2, the highest daily case counts for the Delta wave are lower than the Omicron wave, but the width (length of time) is wider. In addition, the Delta wave seems to occur on top of a previous smaller wave, i.e., from April to June 2021. The Omicron wave result shows that for most of the states, the increasing trend occurred with the disease case counts mostly (if not all) overlapped with the country-level result.

In addition to the two major waves, Figure 8 also indicated some shorter waves that happened from time to time at different states. Similar to the country-level analysis, there are some unstable periods of human activity intensity along with some increasing trends in disease cases that appeared before the Omicron wave. These minor waves of disease situations and unstable human activity conditions were usually ignored in most studies due to the lack of information such as the variants and local policies or the public attitudes—it would be difficult to discuss without the underlying context of the social-spatial information. However, these small events can also provide more detailed information on the trends and signals of disease events, and the calculation framework proposed in this study is able to capture and present the temporal range of the major and minor waves.

To compare the strength of the relationships between cases and check-ins among the 16 states, the number of days in each of the four groups was counted. The four groups were written as the changes of cases dashed the changes of check-ins: (1) up–up indicated the increase in both cases and check-ins in a day, (2) up–down indicated that the cases had increased while the check-ins decreased, (3) down–up indicated that the cases decreased and check-ins increased, and (4) down–down meant both cases and check-ins decreased. The summations of the same directions (up–up and down–down) and the summation of the opposite directions (up–down and down–up) were also calculated. The numbers were converted into percentages by dividing the total number of days (454 days). Table 1 shows the six percentages of the 16 states and Malaysia.

At the country level, around 65.4% (297 days) had an opposite direction for the new case and check-in counts; the cases were increased whereas the check-ins decreased for 34.8% (158 days); for 30.6% (139 days), the case counts decreased and the check-ins increased. Similarly, most states had a high percentage (60–70%) in the opposite direction, indicating that for these cities, COVID-19 cases are negatively correlated with the number of check-in counts in around 60–70% days within the study period. These states included: Kuala Lumpur (71.6%), Putrajaya (60.8%), Selangor (71.8%), Penang (61.7%), Malacca (70.9%), Labuan (63.0%), Johor (71.4%), Negeri Sembilan (62.1%), Kedah (67.8%), Perak (59.0%), and Sabah (59.0%). This means that for most of the time (around 272–318 days), the 11 states had the new case counts and check-in counts in different directions. In general, the down–up group percentage is slightly higher than the up–down group, indicating that the decrease in case counts is usually associated with the increase in check-in counts, and the time length of this situation is longer than the other way around. These states contained relatively higher percentages of urbanized populations. This can explain the immediate changes in the new cases and the intensity of human activity. In an urban area, the spread of the disease is quick, thus the people are more cautious and more responsive to the situation of the disease. On the other hand, the need for human activity (check-ins) in the city was also higher compared to the rural area. Therefore, when the disease situation becomes serious, the reduction in human activity would quickly happen; when the situation is under control and the cases reduced, the intensity of human activity would also quickly restore.

The other five states where the percentages of opposite direction were less than 60%, including Terengganu, Sarawak, Perlis, Pahang, and Kelantan, had a lower urbanization level (in terms of urban population percentage, see Figure 2). Three of the five states, including Terengganu, Pahang, and Kelantan, are located on the east coast of Peninsular Malaysia; Sarawak is in East Malaysia; Perlis is at the northern tip of the west coast of Peninsular Malaysia. The lower percentages in opposite directions indicated a slightly higher percentage of the same directions and thus led to the situation that the negative relationships between the two datasets (new case counts and check-in counts) were less significant. Furthermore, this also suggested that the disease situation is difficult to determine or predict with the human activity intensity, and the policies on human movement restrictions may not be useful in that area. This may be caused by the rural and distant lifestyles that the regular movement was not as dense as in the city and the cohesive interaction of the village community. The distant lifestyles indicated that the intensity of regular human activity (check-ins) was originally low—only for necessary purposes. The cohesive interaction of the village community indicated close relationships between people in the same community, and these close interactions are also a necessary process. Therefore, when a community is infected with a small number of cases, it would rapidly infects most of the people in the community.

**Figure 9:**
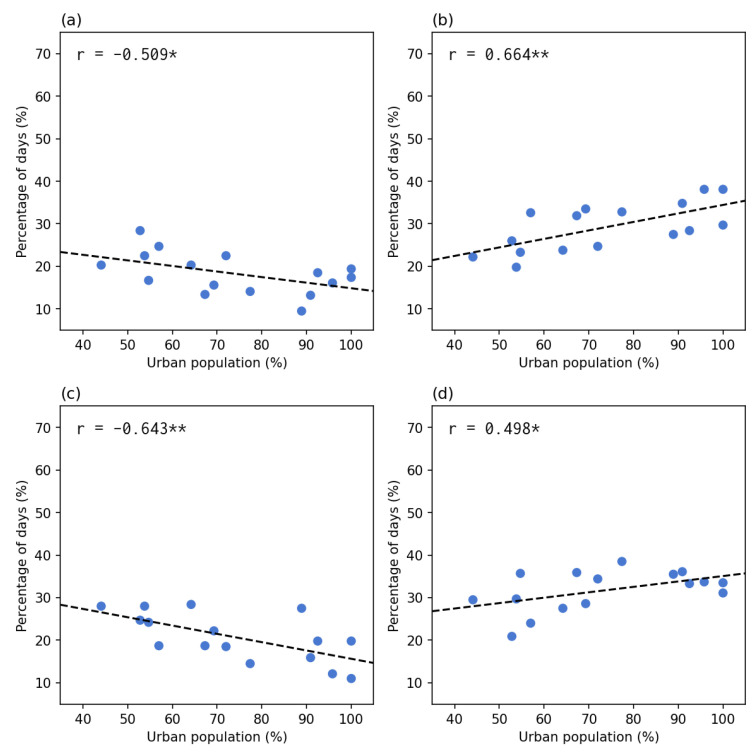

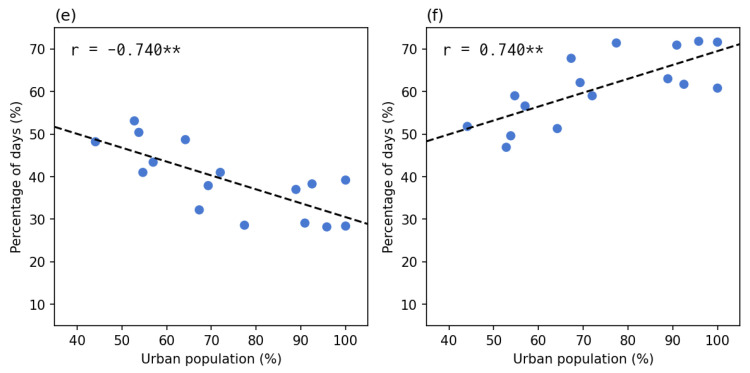
The percentage of days in each of the four groups and the two subtotal groups compared to the percentage of the urbanized population. The left column shows the same direction groups: (**a**) up–up, (**c**) down–down), and (**e**) the subtotal of the same directions. The right column shows the opposite direction groups: (**b**) up–down, (**d**) down–up, and (**f**) the subtotal of opposite directions. Each point indicates a state or federal territory. The correlations were significant at either p≤0.05 (*) level or p≤0.005 (**) level.

Figure 9 shows the correlation between the percentage of the groups of case check-in directions and the percentage of the urbanized population. The result indicated that the same directions groups (left column) all presented a significant negative correlation, whereas the opposite directions groups (right column) presented a significant positive correlation. The correlations were stronger in the last row (the subtotal groups of the same and opposite directions). In other words, the lower the percentage of the urbanized population, the more days the cases and check-ins were both in the increasing or decreasing trend; the higher the percentage of the urbanized population, the more days the cases and check-ins were in the opposite directions (one increase then another decrease). The correlation results confirmed the observation from Figure 8 and Table 1.

## 4. Discussion

This study used the check-in data as a travel willingness to measure the association with recurring pandemic waves. To characterize the trend of check-in data and pandemic waves, we adopted the LMAR method to differentiate the increasing and decreasing tendencies. The results showed that the taking MCO (of any of the MCO series) effect significantly declined people’s travel willingness; however, traveling willingness was resumed after the first relaxing MCO. Remarkably, some people tended to go outside in the second exiting MCO period, even under an increase in confirmed cases, indicating a revenge travel phenomenon. In addition, the impacts of pandemic waves gradually declined travel willingness after September 2021 and disappeared in April 2022.

Based on our findings, we generalized the travel willingness and recurring pandemic waves into a four-stage model: (1) government-imposed compulsory travel restrictions, (2) revenge travel, (3) self-imposed spontaneous epidemic prevention, and (4) new normal. The first stage occurred when the epidemic situation was serious and the mandatory movement restriction or lockdown measures had to be enforced by the government to reduce disease transmission. Therefore, our results showed that the check-in volume rapidly declined as soon as the implementation of MCO 3.0 (around April 2021), consistent with the changes in the urban mobility pattern before and after the travel restriction policy adopted in most countries [30,31]. The second stage is a universal phenomenon observed after a series of compulsory travel restrictions in most countries. Because people are not allowed to have inter-city or international travels for a long time due to the movement restriction measures, the impulsion of people to travel becomes stronger compared to pre-COVID-19, even if the number of confirmed cases is increasing [39,40]. Seçilmiş et al. [41] addressed that this phenomenon was derived from the stimulus-organism-response model since the travel willingness was depressed during a long-term travel restriction. Hence, relaxing travel restrictions would stimulate people’s travel willingness; the travel behavior of friends and family members would also accelerate their travel intention, resulting in making a decision to travel. This also implied that the hedonic behavior could dominate the traveling decision that ignores the epidemic situation [39,42]. The third stage happens when people are getting used to the epidemic situation, including the reduced social activities and the continuing/recurring pandemic waves. People tend to stay at home when there is no restriction or the restriction was relaxed. Although human activity intensity demonstrated an inverse trend with the epidemic situation, our results showed that the changes in travel willingness triggered by the epidemic situation gradually declined, consistent with previous studies [43,44]. This can be caused by diminishing marginal utility. The impulse to travel has declined, and the fear of epidemic situations has been restored. Therefore, a long-term and periodic stimulus could not consistently raise the travel willingness and drive the intention to make a traveling decision [41,43]. The fourth stage is the converging result from the third stage when people adapted to the pandemic. This would lead to a new normal situation in which both the government and people were aware of the response to the confirmed cases and coming variants/waves, and sufficient prevention plans were ready [45].

The inverse patterns between check-in data and pandemic waves in the results of 2021 when the movement restriction was relaxed suggested that strict lockdown measures may not be necessary for a regular wave of the pandemic—the self-imposed movement reduction would naturally happen and would be sufficient for a smaller epidemic wave and a less severe variant of the virus. People may spontaneously be aware of the severity of epidemic situations and take a series of appropriate disease prevention measures to reduce the risks of exposure and infection, as the inverse relationship between check-in data and pandemic waves. In the year 2022, the two datasets started to have a less negative correlation. More overlapping occurred between the two LMAR values. This means that the dynamic trend of the two datasets started to happen in the same direction. Both the human activity intensity and disease situation increased or decreased at the same time. When both increased, it suggested that the people did not care about the disease situation and continued their daily activities. On the other hand, a decreasing trend can be observed at the end of the study period in most states/FTs. This may be caused by the policy changes toward the post-pandemic period—the reopening and recovery stage. People were not following the rules for check-ins since it was planned to be lifted in May 2022. This uncertainty weakens the negative correlations between check-ins and COVID-19 cases.

The conventional time series methods mainly analyze the trend of pandemic waves as a smoothed curve making the identification of the increasing and decreasing periods a difficult task [34,35]. Meanwhile, our results showed that both MACD and LMAR illustrated similar trends in every region. In the comparison between LMAR and MACD, LMAR results are more sensitive to the pattern of the early stage’s daily new case counts compared to the MACD counterpart. Moreover, the increasing and decreasing patterns in LMAR between check-in counts and new case counts are significantly and inversely correlated. Thereby, LMAR is suitable for detecting the new pandemic wave and for facilitating the discussion of travel willingness.

This study has three major limitations: (1) the lack of mobility distance measurements, (2) the low spatial resolution of the disease and check-in data, and (3) the unknown purpose of check-in. The check-in data could not indicate the mobility distance from residential locations to locations of activity. No movement distance information was available. Hence the changes in the movement distance through different stages of the pandemic period can not be explored. Second, the available disease and check-in data were both at the state level. Thus, the impact of the local demographic and economic factors on disease and check-in trends could not be discussed. The only feasible analysis is on the state-level urbanized population percentage as presented. Third, the type of premises was not differentiated in the check-in dataset. Therefore, the influences of the types of activities on travel willingness and disease cases could not be discussed.

## 5. Conclusions

In this study, we demonstrated the calculation of the LMAR using the check-in data, i.e., the surrogate measurement of the human activity intensity and travel willingness, and the confirmed disease cases. We investigated the association between travel willingness and the pandemic waves in Malaysia at the country level and state levels. The recurring pandemic waves could not sustain and stimulate the awareness of infections to maintain low travel willingness. Meanwhile, the gradually declined impact reflected the diminishing marginal benefits effect of pandemic waves on travel willingness and human activity. We generalized the progress of the disease waves and check-in data into a four-stage travel willingness model using the stimulus-organism-response model and diminishing marginal utility: (1) government-imposed movement restrictions, (2) revenge travel, (3) self-imposed movement reduction, and (4) the new normal. The result suggested that when a severe variant and pandemic wave begins, a compulsory movement restriction had to be imposed by the government; if it is a regular wave of a less severe variant, self-prevention measures and self-imposed movement reduction could be sufficient.

## Figures and Tables

**Table 1:** The percentage of days with both increased (up–up), new case count increased and check-ins decreased (up–down), new case count decreased and check-ins increased (down–up), and both decreased (down–down). The percentages of both in the same direction (up–up or down–down) and in the opposite directions (up–down or down–up) were also calculated.

Case Study	Up–Up	Up–Down	Down–Up	Down–Down	Same Direction	Opposite Directions
Malaysia	16.7	30.6	34.8	17.8	34.6	65.4
Kuala Lumpur	17.4	38.1	33.5	11.0	28.4	71.6
Putrajaya	19.4	29.7	31.1	19.8	39.2	60.8
Selangor	16.1	38.1	33.7	12.1	28.2	71.8
Penang	18.5	28.4	33.3	19.8	38.3	61.7
Malacca	13.2	34.8	36.1	15.9	29.1	70.9
Labuan	9.5	27.5	35.5	27.5	37.0	63.0
Johor	14.1	32.8	38.5	14.5	28.6	71.4
Perak	22.5	24.7	34.4	18.5	41.0	59.0
Negeri Sembilan	15.6	33.5	28.6	22.2	37.9	62.1
Kedah	13.4	31.9	35.9	18.7	32.2	67.8
Terengganu	20.3	23.8	27.5	28.4	48.7	51.3
Sarawak	24.7	32.6	24.0	18.7	43.4	56.6
Sabah	16.7	23.3	35.7	24.2	41.0	59.0
Perlis	22.5	19.8	29.7	28.0	50.4	49.6
Pahang	28.4	26.0	20.9	24.7	53.1	46.9
Kelantan	20.3	22.2	29.5	28.0	48.2	51.8

## Data Availability

Data are available in a publicly accessible repository published by the Ministry of Health Malaysia under the Open Data License: https://github.com/MoH-Malaysia/covid19-public (accessed on 28 October 2022), detail of open data license: https://www.data.gov.my/p/pekeliling-data-terbuka (accessed on 18 January 2023).

## Abbreviations

The following abbreviations are used in this manuscript:

MACD	Moving Average Convergence/Divergence
MCO	Movement Control Order
NRP	National Recovery Plan
STMA	Short-term Moving Average
LTMA	Long-term Moving Average
LMAR	Log Moving Average Ratio
FT	Federal Territory
